# Mental health of patients with adolescent idiopathic scoliosis and their parents in China: a cross-sectional survey

**DOI:** 10.1186/s12888-019-2128-1

**Published:** 2019-05-14

**Authors:** Hai Wang, Tao Li, Wangshu Yuan, Zheping Zhang, Jing Wei, Guixing Qiu, Jianxiong Shen

**Affiliations:** 1 Department of Orthopaedic Surgery, Peking Union Medical College Hospital, Peking Union Medical College and Chinese Academy of Medical Sciences, Beijing, China; 2 Department of Psychology, Peking Union Medical College Hospital, Peking Union Medical College and Chinese Academy of Medical Sciences, Beijing, China; 3 Department of Rehabilitation, Peking Union Medical College Hospital, Peking Union Medical College and Chinese Academy of Medical Sciences, Beijing, China; 4 Department of Orthopaedic Surgery, Beijing Puren Hospital, Beijing, China

**Keywords:** Adolescent idiopathic scoliosis (AIS), Parents, Depression, Anxiety, Mental health

## Abstract

**Backgrounds:**

Adolescent idiopathic scoliosis (AIS) is an adolescent onset spinal deformity, which can negatively affect the mental health of these patients. But no studies about their parental mental health have been reported so far. In this study, the parental mental health of AIS patients and the associated risk factors were evaluated by a cross-sectional survey.

**Methods:**

64 AIS patients who underwent conservative or surgical treatments in our hospital from April 2017 to March 2018, and their parents were enrolled in the AIS group. 85 parents of healthy children were enrolled in the control group. Depression and anxiety were separately assessed using the Patient Health Questionnaire (PHQ-9) and the Generalized Anxiety Disorder 7-item scale (GAD-7). Spearman correlation coefficients were first estimated to investigate the relationships among the parental PHQ-9/GAD-7 scores and the patient’s PHQ-9/GAD-7 scores in the AIS group. Then, the morbidities of the parental probable major depressive disorder (pMDD, PHQ score ≥ 10) and probable general anxiety disorder (pGAD, GAD-7 score ≥ 10) were compared between the AIS and control groups. Third, the potential risk factors for parental pMDD or pGAD in the AIS group were compared using the chi-squared test or Student’s t-test, respectively. Finally, the uneven distributive variates were analyzed using the binary logistic regression model.

**Results:**

Both parental depression and anxiety were moderately associated with those of the patients (r = 0.448~0.515, *p* < 0.01) in the AIS group, respectively. The morbidities of parental pMDD and pGAD in the AIS group were 14.1%, significantly higher than those in the control group (pMDD = 4.7%, *p* = 0.045; pGAD = 3.5%, *p* = 0.019). A Cobb angle of the major curve ≥50° (*p* = 0.034, odds ratio [OR] = 8.264), patients with pMDD (*p* = 0.018, OR = 17.576), and low education level of the parents (*p* = 0.026, OR = 0.122) were the risk factors of parental pMDD. Household income < 8000 rmb/month was the risk factor for parental pGAD (*p* = 0.021).

**Conclusions:**

The morbidities of pMDD and pGAD in parents of AIS patients were higher than those in parents of healthy children. Parental depression and anxiety were closely associated with their children’s depression and anxiety. Therefore, the parental mental health of AIS patients should be paid attention to, especially for those parents with risk factors.

## Backgrounds

Scoliosis is a three dimensional deformation of the spine with a Cobb angle of the major curve ≥10° in a standing anteroposterior X-ray image [1]. About 85% of these individuals are idiopathic scoliosis with no known etiology [2]. According to the age of onset, idiopathic scoliosis can be classified as infantile, juvenile, and adolescent [1]. Among them, adolescent idiopathic scoliosis (AIS) is the most common type, with a prevalence rate of approximately 2 to 3% based on data from the school screening studies [3, 4].

AIS can negatively affect the mental health of the patients, especially resulting in depression [5]. Several instruments, such as the Short-Form-36(SF-36), the Bad Sobernheim Stress Questionnaire (BSSQ), and the Scoliosis Research Society 22 Questionnaire (SRS-22), have been used to assess the mental health of AIS patients [6, 7]. Their parents, the close family caregivers, might also be affected because they have to take on new responsibilities, or give up past activities due to the stress of their children’s deformities and mental health alterations. Several previous studies have confirmed that the parents of the AIS patients may also be affected by this deformity [8–13]. The parental anxiety level of AIS patients decreased significantly from preoperative to postoperative stages but still remained high [13]. The parents view the deformity differently from the patient, as they overestimate the patient’s stress level, and have greater concerns and expectations regarding scoliosis surgery [8, 11, 12].

Common mental disorders (CMDs) such as depression and anxiety can have implications for long-term health outcomes associated with increased fatigue, impaired long-term disease activity and physical disability [14]. Therefore, it is very important for scoliosis surgeons to recognize and address parental CMDs when parents accompany the AIS patient to see a doctor. In this study, we hypothesized that the parental mental health of patients with AIS might be negatively affected and related to some potential risk factors, such as patients’ factors, socio-economic factors, and the severity of the deformity.

## Materials and methods

### Samples

This cross-sectional descriptive study was conducted in the Department of Orthopaedic Surgery and Department of Rehabilitation in the authors’ hospital, between April, 2017 and March, 2018. A total of 64 consecutive AIS patient (aged 11–18 years old)-parent pairs were enrolled in this study. All the AIS patients were diagnosed by two spine deformity surgeons based on their medical history and images. There were 5 males and 59 females in the patient group and 17 fathers and 47 mothers in the parent group. A total of 85 parents (23 fathers and 62 mothers) of health children with age of 11–18 years old was enrolled in the control group. All the participants were orally informed, and the parents gave their written consent.

### Procedures

All the questionnaires were administered and data collected before operation or physiotherapy. Questions about socio-demographic data including household income, marital status, employment, and education were answered by parents. Brace history was defined as “yes”, referring to Misterska’s report [12], if a patient ever underwent the brace application for at least 12 h a day for more than 1 month before the survey. The deformity data were measured by two experienced surgeons based on the X-ray images, including the Cobb angle of the major curve, frontal balance, and shoulder height.

### Instruments

Depression and anxiety of all the participants were separately evaluated using a nine-item Patient Health Questionnaire (PHQ-9) and a seven-item Generalized Anxiety Disorder scale (GAD-7) in Chinese.

The PHQ-9 is a widely used self-report screening tool for depression based on the Diagnostic and Statistical Manual of Mental Disorders-IV (DSM-IV) criteria [15]. The tool consists of 9 items that assess whether the symptoms have bothered the individual during the previous 2 weeks. The summed score ranges from 0 to 27, and can be categorized into 4 categories: minimal (0~4), mild (5~9), moderate (10~14), and severe (≥15). In most studies, a cutoff point of 10 has been accepted for the diagnostic criteria of probable major depressive disorder (pMDD) [16]. Its pooled sensitivity and specificity were 0.78 and 0.87, respectively. The reliability and validity of its Chinese version has been confirmed previously [17].

The GAD-7 has been shown to be a valid and effective measure of anxiety in the general population [18]. The item scales of this measure, based on DSM-IV criteria, focus on the presence of 7 core anxiety symptoms in the last 2 weeks. The total scores range between 0 and 21, and can also be categorized into 4 categories: minimal (0~4), mild (5~9), moderate (10~14), and severe (≥15). A cutoff point of 10 has been set for diagnosing probable generalized anxiety disorder (pGAD) [19]. Its sensitivity and specificity were 0.89 and 0.82, respectively. The reliability and validity of its Chinese version have also been confirmed previously [17].

### Statistics

Means ± SDs and ranges of values or proportions were separately used to describe the measurement data and the categorical data. Spearman correlation coefficients were estimated in order to investigate the associations between the parental PHQ-9 scores and GAD-7 scores and PHQ-9/GAD-7 scores of the patients and their parents if the scores did not fit normal distribution. For the potential risk factors of the parental pMDD (PHQ-9 ≥ 10) or pGAD (GAD-7 ≥ 10), group differences were first analyzed using the chi-squared test for the categorical data (sex of patients, Cobb angle of the major curve, planned surgery, pMDD of patients, pGAD of patients, shoulder height, brace treatment, parenthood, education of parents, employment of parents, health insurance, residence, parental marriage, live with parents, and household income) and the independent t-test for the measurement data (age of patients, age of parents, course of disease, and frontal balance). The effects of independent risk factors on pMDD or pGAD were analyzed using the logistic regression model. The data were analyzed using SPSS, version 22.0 (IBM Corp., Armonk, New York, USA). *P* < 0.05 was considered statistically significant.

## Results

### General characteristics

A total of 64 AIS patients (age = 14.3 ± 2.2 years old; male: female = 5:59) and their parents (age = 42.4 ± 4.3 years old; male: female =17:47) were enrolled in this study. The mean Cobb angle of the major curve was 44.2 ± 18.9°. The general characteristics of the included samples are summarized in Table 1.

**Table 1:** The general characteristics of the samples (64 patient-parent pairs)

Variables	Value
Age of patients (years old)	14.3 ± 2.2
Sex of patients Male	5 (7.8%)
Female	59 (92.2%)
Brace application Yes	19 (29.7%)
No	45 (70.3%)
Cobb angle of the major curve(°)	44.2 ± 18.9
Frontal balance (mm)	11.2 ± 10.1
Shoulder height Equal	16 (25.0%)
Unequal	48 (75.0%)
Age of parents (years old)	41.7 ± 3.7
Sex of parents Male	17 (26.6%)
Female	47 (73.4%)
Health insurance (Yes)	51 (79.7%)
Residence City	55 (85.6%)
Countryside	9 (14.4%)
Marriage of parents Married	58 (90.6%)
Divorced	5 (7.8%)
Widowed	1 (1.6%)
Live with parents Yes	61 (95.3%)
No	3 (4.7%)
Household income ≤4000 rmb/month	17 (26.6%)
4000~8000 rmb/month	25 (39.1%)
≥8000 rmb/month	22 (34.4%)
Employment of father Yes	53 (84.1%)
No	10 (15.9%)
Employment of mother Yes	40 (62.5%)
No	24 (37.5%)
Education of father Primary school	1 (1.6%)
Junior high school	11 (17.5%)
Senior high school	19 (30.2%)
University	32 (50.8%)
Education of mother Primary school	1 (1.6%)
Junior high school	19 (29.7%)
Senior high school	20 (31.3%)
University	24 (37.5%)

*rmb* Renminbi

### PHQ-9 scores and GAD-7 scores

The mean PHQ-9 and GAD-7 scores of the AIS patients were 4.0 ± 4.0 (0~16) and 2.9 ± 3.5 (0~19), respectively, and the parental mean scores were 4.7 ± 5.0 (0~20) and 4.2 ± 4.7 (0~19), respectively. Table 2 shows the distribution of PHQ-9 scores and GAD-7 scores for patients and their parents. There were more parents with severe depressive or anxious symptoms (≥15) than patients.

**Table 2:** PHQ-9 scores and GAD-7 scores of the patients and their parents

	Minimal (0~4)	Mild (5~9)	Moderate (10~14)	Severe (≥15)
Patients				
PHQ-9 score	39 (60.9%)	20 (31.3%)	4 (6.3%)	1 (1.6%)
GAD-7 score	49 (76.6%)	13 (20.3%)	1 (1.6%)	1 (1.6%)
Parents				
PHQ-9 score	39 (60.9%)	16 (25.0%)	4 (6.3%)	5 (7.8%)
GAD-7 score	44 (68.8%)	11 (17.2%)	5 (7.8%)	4 (6.3%)

According to the definition of pMDD (PHQ-9 ≥ 10), the morbidities were 7.9% (5/64) and 14.1% (9/64) for the AIS patients and their parents in the AIS group, respectively. The morbidity of the parental pMDD in the AIS group was significantly higher than that in the control group (14.1% vs 4.7%, *p* = 0.045, Table 3). Similarly, the morbidities of pGAD (GAD-7 ≥ 10) were 3.2% (2/64) and 14.1% (9/64) for the AIS patients and their parents in the AIS group, respectively. The morbidity of the parental pGAD in the AIS group was significantly higher than that in the control group (14.1% vs 3.5%, *p* = 0.019, Table 3). A total of 17.2% (11/64) parents in the AIS group suffered from pCMDs, and approximately 77.8% (7/9) of parents with pMDD were also diagnosed with pGAD.

**Table 3:** The comparative test results of the parental mental health between the AIS group and the control group

Variables	AIS(*n* = 64)	Control(*n* = 85)	*p*
Parental Age (years old)	42.5 ± 4.3	41.9 ± 4.6	0.420
Sex Male	17 (26.6%)	23 (27.1%)	0.946
Female	47 (73.4%)	62 (72.9%)	
pMDD Yes	9 (14.1%)	4 (4.7%)	0.045
No	55 (85.9%)	81 (95.3%)	
pGAD Yes	9 (14.1%)	3 (3.5%)	0.019
No	55 (85.9%)	82 (96.5%)	

### Relationships between PHQ-9 and GAD-7 scores in the AIS group

The PHQ-9 scores of the parents were strongly correlated with their GAD-7 scores of the parents (r = 0.690, *p* < 0.01, Table 4). The PHQ-9 scores of the parents were moderately correlated with the PHQ-9 scores and the GAD-7 scores of the patients (r = 0.499 and 0.448, respectively; *p* < 0.01, Table 4). Additionally, the GAD-7 scores of the parents were also moderately associated with the PHQ-9 scores and the GAD-7 scores of the patients (r = 0.515 and 0.469, respectively; *p* < 0.01, Table 4). Upon further comparison, the maternal PHQ-9 scores and GAD-7 scores were higher than the paternal scores, but the difference was only significant for the PHQ-9 scores (PHQ-9 scores:5.5 ± 5.3 vs 2.5 ± 2.8, *p* = 0.005; GAD-7 scores: 4.5 ± 5.1 vs 3.4 ± 3.1, *p* = 0.403).

**Table 4:** Spearman Correlation analysis of PHQ-9 scores and GAD-7 scores in the AIS group

	Parents	
	PHQ-9 score	GAD-7 score
Parents		
PHQ-9 score	–	0.690**
GAD-7 score	0.690**	–
Patients		
PHQ-9 score	0.499**	0.515**
GAD-7 score	0.448**	0.469**

**significant at the level of *p* < 0.01

### Risk factors for parental pMDD in the AIS group

All the potential risk factors were first compared between the parents with and without pMDD using the chi-squared test or the independent t-test. As shown in Table 5, a Cobb angle of the major curve≥50゜, pMDD in patients, low education level of the parents, and household income< 8000 rmb/month were significantly associated with parental pMDD (*p* = 0.022, 0.017, 0.022, and 0.021, respectively). Further, the binary logistic regression results revealed that a Cobb angle of the major curve≥50゜ (*p* = 0.034, odds ratio [OR] = 8.264), pMDD of patients (*p* = 0.018, OR = 17.576), and low education level of the parents (*p* = 0.026, OR = 0.122) were the independent risk factors for parental pMDD (Table 6).

**Table 5:** Univariate analysis results of parental pMDD in the AIS group

Variables	PHQ-9 score		*p*
	< 10(*n* = 55)	≥10(*n* = 9)	
Age of patients (years old)	14.3 ± 2.1	14.8 ± 2.6	0.511
Course of disease (months)	18.5 ± 20.5	24.2 ± 27.8	0.464
Frontal Balance (mm)	11.0 ± 10.3	13.0 ± 8.8	0.575
Major Curve < 50°	37 (94.9%)	2 (5.1%)	0.022*
≥50°	18 (72.0%)	7 (28.0%)	
Sex of patients Male	3 (60.0%)	2 (40.0%)	0.141*
Female	52 (88.1%)	7 (11.9%)	
Planed surgery Yes	29 (82.9%)	6 (17.1%)	0.494*
No	26 (89.7%)	3 (10.3%)	
MDD of patients No	53 (89.8%)	6 (10.2%)	0.017*
Yes	2 (40.0%)	3 (60.0%)	
GAD of patients No	54 (87.1%)	8 (12.9%)	0.263*
Yes	1 (50.0%)	1 (50.0%)	
Shoulder height Equal	14 (87.5%)	2 (12.5%)	1.000*
Unequal	41 (85.4%)	7 (14.6%)	
Brace treatment Yes	15 (78.9%)	4 (21.1%)	0.432*
No	40 (88.9%)	5 (11.1%)	
Age of parents	42.7 ± 4.3	41.0 ± 3.8	0.270
Parenthood Father	16 (94.1%)	1 (5.9%)	0.424*
Mother	39 (83.0%)	8 (17.0%)	
Education of parents Primary/Junior school	14 (70.0%)	6 (30.0%)	0.022*
Senior school/University	41 (93.2%)	3 (6.8%)	
Job of parents Yes	37 (88.1%)	5 (11.9%)	0.480*
No	18 (81.8%)	4 (18.2%)	
Health Insurance Yes	44 (86.3%)	7 (13.7%)	1.000*
No	11 (84.6%)	2 (15.4%)	
Residence City	48 (87.3%)	7 (12.7%)	0.602*
Countryside	7 (77.8%)	2 (22.2%)	
Parental Marriage Married	49 (84.5%)	9 (15.5%)	0.582*
Divorced/widowed	6 (100.0%)	0 (0.0%)	
Live with parents Yes	53 (86.9%)	8 (13.1%)	0.370*
No	2 (66.7%)	1 (33.3%)	
Income < 8000 rmb/month	32 (78.0%)	9 (22.0%)	0.021*
≥8000 rmb/month	23 (100.0%)	0 (0.0%)	

* Fisher exact test

**Table 6:** Binary logistic regression results of parental pMDD (Method = LR)

Variables	*p*	Odd ratio (OR)	95% confidence interval (CI)
Cobb angle of the major curve≥50°	0.034	8.264	1.178~57.947
MDD of patients	0.018	17.576	1.619~190.772
Education of parents	0.026	0.122	0.019~0.780
Household income	0.051	< 0.001	< 0.001~ > 999.999

### Risk factors for parental pGAD in the AIS group

First, the potential risk factors were also first compared between the parents with and without pGAD using the chi-squared test or the independent t-test. The results showed that only household income< 8000 rmb/month was significantly associated with parental pGAD (*p* = 0.021, Table 7). Therefore, it was not necessary to conduct a binary logistic regression test to eliminate the influences of farraginous factors. Household income< 8000 rmb/month was the independent risk factor for parental pGAD.

**Table 7:** Univariate analysis results of parental pGAD in the AIS group

Variables	GAD-7 score		*p*
	< 10(*n* = 55)	≥10(*n* = 9)	
Age of patients (years old)	14.2 ± 2.1	15.0 ± 2.5	0.325
Course of disease (months)	18.5 ± 20.5	24.6 ± 27.5	0.434
Frontal Balance (mm)	11.2 ± 10.3	11.7 ± 8.8	0.891
Major Curve < 50°	36 (92.3%)	3 (7.7%)	0.137*
≥50°	19 (76.0%)	6 (24.0%)	
Sex of patients Male	3 (60.0%)	2 (40.0%)	0.141*
Female	52 (88.1%)	7 (11.9%)	
Planed surgery Yes	29 (82.9%)	6 (17.1%)	0.494*
No	26 (89.7%)	3 (10.3%)	
MDD of patients No	52 (88.1%)	7 (11.9%)	0.141*
Yes	3 (60.0%)	2 (40.0%)	
GAD of patients No	53 (85.5%)	9 (14.5%)	1.000*
Yes	2 (100.0%)	0 (0.0%)	
Shoulder height Equal	15 (93.8%)	1 (6.2%)	0.430*
Unequal	40 (83.3%)	8 (16.7%)	
Brace treatment Yes	16 (84.2%)	3 (15.8%)	1.000*
No	39 (86.7%)	6 (13.3%)	
Age of parents	42.3 ± 3.9	43.8 ± 6.4	0.326
Parenthood Father	16 (94.1%)	1 (5.9%)	0.424*
Mother	39 (83.0%)	8 (17.0%)	
Education of parents Primary/Junior school	15 (75.0%)	5 (25.0%)	0.124*
Senior school/University	40 (90.9%)	4 (9.1%)	
Job of parents Yes	38 (90.5%)	4 (9.5%)	0.254*
No	17 (77.3%)	5 (22.7%)	
Health Insurance Yes	44 (86.3%)	7 (13.7%)	1.000*
No	11 (84.6%)	2 (15.4%)	
Residence City	48 (87.3%)	7 (12.7%)	0.602*
Countryside	7 (77.8%)	2 (22.2%)	
Parental Marriage Married	50 (86.2%)	8 (13.8%)	1.000*
Divorced/widowed	5 (83.3%)	1 (16.7%)	
Live with parents Yes	53 (86.9%)	8 (13.1%)	0.370*
No	2 (100.0%)	1 (0.0%)	
Income < 8000 rmb/month	32 (78.0%)	9 (22.0%)	0.021*
≥8000 rmb/month	23 (100.0%)	0 (0.0%)	

* Fisher exact test

## Discussion

In this study, we were able to depict a remarkable increase in the incidence of pCMDs (pMDD and pGAD) prevalence among the parents of AIS patients. Morbidities of both parental pMDD and pGAD in the AIS group were significantly higher than those in the control group. Previously, AIS has been regarded as a chronic condition that affects an adolescent’s bodily configuration, consequently leading to alterations in psychological health and lifestyle [5, 7]. The parents of these patients, the closest and long-term caregivers, might be forced to make changes in their own lives, take on new responsibilities, or give up past activities due to the stress of their children’s illness and alterations. The Caregiving Career/Stress Process model suggests that life events can lead to persistent negative changes in people’s roles that wear away at desirable elements of self-concept with the arousal of stress [20, 21]. Once the demands and obstacles that exceed or push parental capacity to adapt to the limit, the mental health of the parents can be negatively affected. The relationship between the parental perception of how difficult it is to care for the child and the feelings of depression has been confirmed and might explain why the parental anxiety of AIS patients still remains high after surgery [13, 22].

In the clinic, deformity surgeons should pay attention to the parental mental health of AIS patients when diagnosing and treating these patients. Because CMDs can not only deteriorate the quality of life and social functioning but also cause the development and exacerbation of chronic illness [23]. It has been confirmed that cardiovascular disease, diabetes, asthma, smoking, and obesity are all significantly associated with anxiety and depression [24].

Depression and anxiety in the parents shared a very close relationship. More than half of the parents diagnosed with pMDD also had pGAD. Depression and anxiety almost always go together, as depression tends to be past-oriented, whereas anxiety is future-oriented [25, 26]. Therefore, we should meanwhile focus on depression and anxiety in parents.

Both depression and anxiety in the parents were moderately associated with those in the patients, respectively. There might be two reasons why the psychiatric symptom scores of the parents were associated with similar psychopathology in their children: the heritability and/or the parenting stress [27]. Compared to the mental health status of the fathers, maternal mental health was more likely to be elevated, especially for depression. The PHQ-9 scores of the mothers were significantly higher than those of the fathers. Because caregiver stress tends to be higher in mothers, maternal depression is more strongly associated with internalizing problems in children than paternal depression [28, 29].

More insight into these risk factors of pMDD or pGAD can facilitate earlier recognition of these parents that may benefit from more intensive treatment. As reported previously, general risk factors for parental psychopathology include financial problems, unemployment, divorce, being a single parenting, and the demographic characteristics of the parent and child [30, 31]. Referring to Raina’s multidimensional model for examining the determinants of psychological health in the parents of children with health issues [21], these sociodemographic variables and some clinical variables were selected as potential risk factors for pMDD or pGAD to analysis in this study. The results showed that a Cobb angle of the major curve ≥50°, patients with pMDD, and low education level of parents were the risk factors for parental pMDD; household income < 8000 rmb/month was the risk factor for parental pGAD.

Parental pMDD was associated with a Cobb angle of the major curve ≥50°. A higher Cobb angle of the major curve indicates the possibility of disturbed appearances, curve progression, and severe back pain. All these concerns might arouse or aggravate the parental depression. Otherwise, surgical procedures are often be suggested when the Cobb angle of the major curve is more than 50°. Although planed surgery is not a risk factor for pMDD, it is possible that the fear and burden of surgery may further aggravate the parental depression based on these above mentioned concerns.

Parental pMDD was strongly correlated with patients with pMDD. Parents whose children suffer from psychiatric symptoms are at risk for psychiatric symptoms themselves [26]. As discussed above, parental depression can be affected by their offspring’s depression through two pathways: the heritability and/or the parenting stress [27]. Moreover,parents’ perceptions of their child’s depression regarding their deformity are stronger than their child’s own assessment [12].

Low education level of the parents was an independent risk factor for parental pMDD. There is an inverse association between education attainment and depression, as reported previously [32]. In addition, the protective effect of education will increase somewhat with time throughout one’s life [33].

Low household income (≤8000 rmb/month) was the only risk factor for parental pGAD in this study. A similar relationship has been reported between low levels of household income and CMDs, including GAD [34]. This might be explained by social causation and social selection [35]. Social causation posits that adversity, stress, and reduced capacity to cope, as they relate to low income increase the risk of CMDs. The social selection hypothesis suggests that individuals with CMDs have a predisposition to declining socioeconomic status.

These findings support the recognition that the parents of children with AIS may suffer from CMDs and suggest the importance of conducting assessments of the parental psychosocial support needs. According to these risk factors, the screening need of the parents can be classified at two levels. It may help to first identify vulnerable parents, especially for those with risk factors, and then facilitate early referral for more focused assessment and interventions.

There were several limitations in this study. First, the sample size was limited, because it was a single center study. Second, we have not studied parents’ coping strategies for meeting the stress of their children’s illness. Future studies should be conducted with an objective study to research and discuss these coping factors.

## Conclusions

The morbidities of pMDD and pGAD in parents of children with AIS were higher than those in parents with healthy children. Parental depression and anxiety were closely associated with their child’s depression and anxiety. A Cobb angle of the major curve ≥50°, patients with pMDD, and low education level of the parents were the independent risk factors for parental pMDD. Low household income was the risk factor for parental pGAD. The parental mental health status of AIS patients should be paid attention to by deformity surgeons, especially for those parents with risk factors, it would help to facilitate early referral for more focused assessment and interventions. Insights into the parental coping strategies can help to guide treatment in the future research.

## Abbreviations

95%CI: 95% Confidence Interval
AIS: Adolescent Idiopathic Scoliosis
BSSQ: Bad Sobernheim Stress Questionnaire
CMDs: Common Mental Disorders
GAD-7: Seven-item Generalized Anxiety Disorder Scale
OR: Odds Ratio
pGAD: Probable Generalized Anxiety Disorder
PHQ-9: Nine-item Patient Health Questionnaire
pMDD: Probable Major Depressive Disorder
SF-36: Short-Form-36
SRS-22: Scoliosis Research Society 22 Questionnaire
